# Reproductive health in humanitarian settings in Lebanon and Iraq: results from four cross-sectional studies, 2014–2015

**DOI:** 10.1186/s13031-019-0210-4

**Published:** 2019-06-10

**Authors:** Marta A. Balinska, Robin Nesbitt, Zeina Ghantous, Iza Ciglenecki, Nelly Staderini

**Affiliations:** 1 Médecins Sans Frontières Switzerland, Rue de Lausanne 78, 1211 Geneva, Switzerland; 2 Médecins Sans Frontières Switzerland, P. O. Box 527, Nhlangano, Swaziland; 3 Médecins Sans Frontières France, Genifor Center, Clémenceau Street, Bloc C, Beirut, Lebanon

**Keywords:** Refugees, Family planning, Pregnancy, unplanned, Prenatal care, Delivery, obstetric, Cesarean section, Iraq, Lebanon, Syria

## Abstract

**Background:**

Reproductive health is an important component of humanitarian response. Displaced women need access to family planning, antenatal care, and the presence of a skilled birth attendant at delivery. Since the beginning of the Syrian conflict in 2011, Lebanon and Iraq have been hosting large numbers of refugees, thereby straining local capacities to provide these services. In order to identify salient health needs, Médecins Sans Frontières conducted a survey in several sites hosting refugees and internally displaced persons across the region. Here we describe the reproductive health profile of Syrian refugees, Iraqi displaced persons, and vulnerable Lebanese and their use of services.

**Methods:**

We conducted four cross-sectional surveys in 2014–2015 in two sites in Lebanon and two sites in Iraq. Depending on the site, two-stage cluster sampling or systematic sampling was intended, but non-probability methods were employed at the second stage due to implementation challenges. We collected information on overall health (including reproductive health) and demographic information from heads of households on the basis of a standardized questionnaire. Pearson chi-square tests were used to compare proportions, and generalized linear models were used to calculate odds ratios with regard to risk factors. All analyses were performed using the survey suite of commands in Stata version 14.1.

**Results:**

A total of 23,604 individuals were surveyed, including 5925 women of childbearing age. Overall, it was reported that 7.5% of women were currently pregnant and 12.8% had given birth within the previous 12 months. It was reported that pregnancy was unplanned for 57% of currently pregnant women and 66.7% of women who had delivered in the previous year. A slight majority of women from both groups had accessed antenatal care at least once. Amongst women who had delivered in the previous year, 84.5% had done so with a skilled birth attendant and 22.1% had had a cesarean section. Location and head of household education were predictors of unplanned pregnancy in multivariable analysis. Head of household education was also significantly associated with higher uptake of antenatal care.

**Conclusions:**

Considering the large number of pregnant women and women having recently delivered in these settings, addressing their sexual and reproductive health needs emerges as a crucial aspect of humanitarian response. This study identified unmet needs for family planning and high cesarean section rates at all sites, suggesting both lack of access to certain services (contraception, antenatal care), but also over-recourse to cesarean section. These specific challenges can impact directly on maternal and child health and need today to be kept high on the humanitarian agenda.

## Background

Sexual and reproductive health (SRH) is a challenging issue in the Middle East and the subject has attracted relatively little academic attention over the past 40 years. As a result, the SRH profile of women living in the Middle East, and specifically Syrian women, is not fully described. Before the conflict, Syria enjoyed a well-functioning health system [1]. A survey had shown that 42% of women aged 15 to 49 years were using modern contraceptives [2]. The average fertility rate at the time was 3.8, with around a third of pregnancies stated to be unintended (whether unplanned or unwanted) [3]. Termination of pregnancy (ToP) was allowed only if the mother was at risk of death [4], and the rate of induced abortion was estimated at 3.9% in 2006, although previous studies had found much higher figures [3]. Overall trends in SRH are similar in neighboring countries such as Lebanon, Iraq, and Jordan, where many Syrian refugees are currently living [5–10].

In a conflict and refugee context, it is well known that women and children are at highest risk of adverse health outcomes [1]. In March 2015, the United Nations Population Fund estimated that nearly half a million Syrian women (both in Syria and hosting countries) were pregnant and that more than 70,000 of them would experience complications associated with pregnancy and/or delivery [11]. Many Syrian families (approximately 5.3 million registered refugees) have relocated to neighboring countries, primarily Turkey (3.3 million), Lebanon (1.5 million), Jordan (655,000), Egypt (500,000), and Iraq (246,000) according to the latest available figures [12].

Specifically in Lebanon, due to the continuous influx of displaced people and refugees, the existing health structures were extremely burdened [13]. Gaps in critical health services included key areas such as SRH. Since November 2011, Médecins Sans Frontières (MSF) has been providing free primary care assistance to Syrian refugees in a number of locations in Lebanon, including the treatment of acute and chronic diseases and reproductive health. These services are offered to Syrian refugees (irrespective of registration status), Lebanese and Palestinian refugees from Syria, and the Lebanese host community.

In Iraq, it is widely known that health services are under stress [14, 15], but to our knowledge no specific study has systematically investigated this problem.

**Fig. 1:**
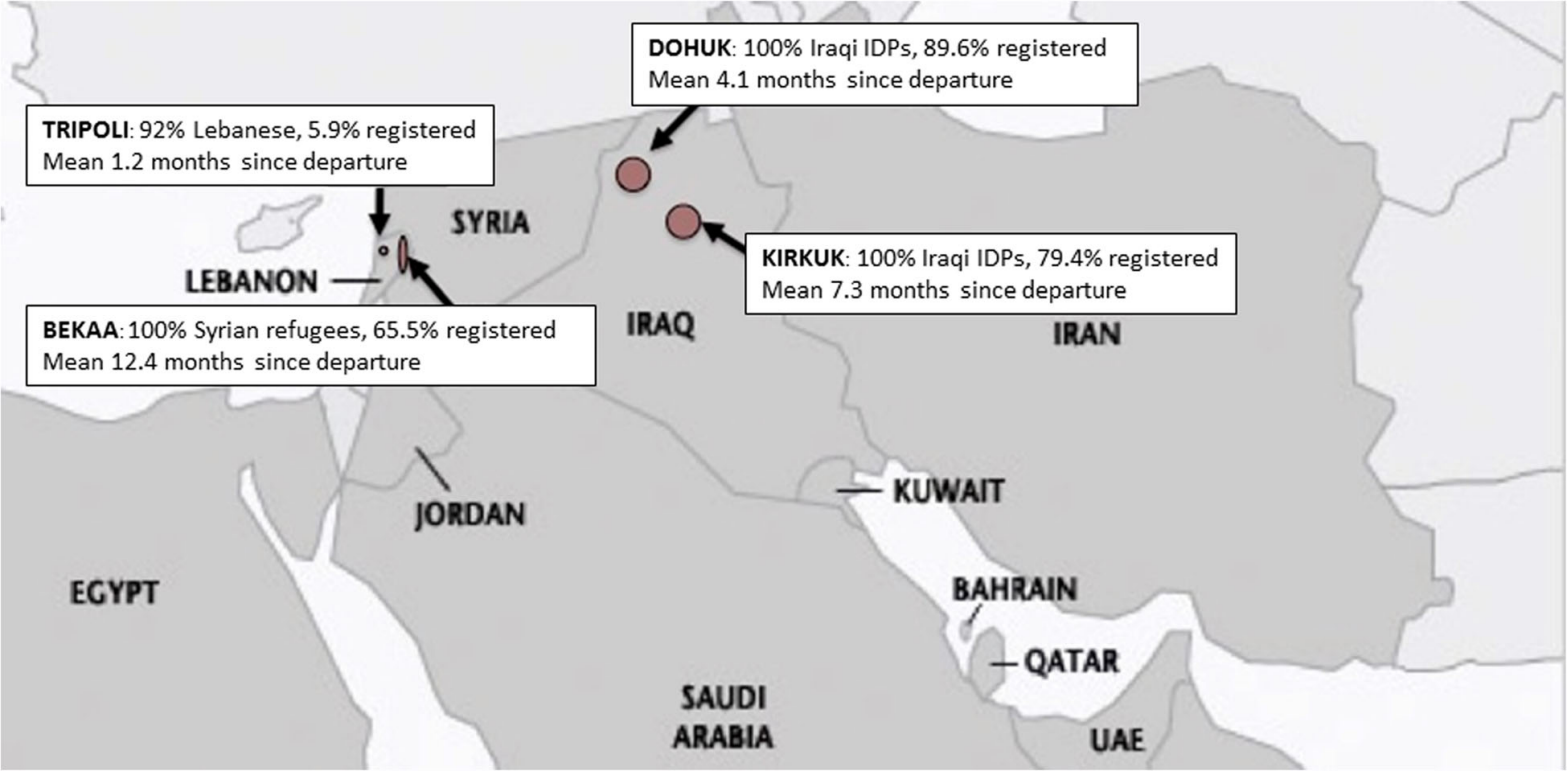
Map of the survey region

In 2014, concerned notably by these populations’ needs for greater care of chronic conditions, MSF conducted a general health assessment in a selection of its project sites both in Lebanon and Iraq where the organization was also operating to help Syrian refugees and Iraqi displaced persons. Here we describe the reproductive health profile of all women of reproductive age (in surveyed households) in Bekaa and Tripoli (Lebanon), and Kirkuk and Dohuk (Iraq), their use of services, and their most salient needs. For a map of the region, see Fig. 1.

## Methods

Four cross-sectional surveys were conducted between February 2014 and April 2015 in four sites (Bekaa, Tripoli, Kirkuk, and Dohuk). Our surveys looked at three different study populations (Syrians, Iraqis, Lebanese). In Bekaa, we wished to sample from the Syrian refugees who had migrated from Syria to Lebanon within the 3 years prior to the survey. In Tripoli, the sample included mainly the hosting Lebanese population who were neither refugees nor IDPs, but did include also some Syrians. In Kirkuk and Dohuk (Iraq), our sample was taken among those displaced during the second wave of displacement which had occurred 12 months prior to the survey. Setting also differed by site with populations living in camps, informal tented settlements, and houses/apartments in both urban and rural areas as follows: a) in Bekaa the majority of respondents lived in urban and rural areas of Baalbek, West Baalbek, Arsal, Zahle, Bar Elias, Al Marj, Majda Anjar, and surrounding villages with some respondents in informal tented settlements, b) in Tripoli respondents were in the urban settings of Bab al Tabanneh and Jabal Mohsen; c) in Kirkuk respondents were living in Kirkuk city and the surrounding areas, or the Yahyawah and Laylan camps, and d) in Dohuk city and surrounding areas respondents residing in as well as in informal tented settlements and camps.

### Survey and sampling methodology

#### Sampling

The survey was designed to utilize two-stage cluster sampling in three of the survey sites (Bekaa, Dohuk, Kirkuk), which included both camps and informal tented settlements. In each site, clusters were identified from sampling frames based on existing data from UNHCR, UNICEF or other NGOs active in the area, and were sampled with probability proportional to size. Systematic sampling was attempted in the second stage; however, due to logistical constraints it is possible that non-probability sampling methods, such as convenience sampling were used at some clusters to identify the first dwelling in the second stage of sampling. In Tripoli, an attempt was made to systematically sample households in the urban communities of Bab al Tabanneh and Jabal Monsen. However, non-probability sampling methods may have been used to identify the first household. Sampling intervals differed by site, and the interviewers proceeded to every fourth (Tripoli) or sixth dwelling (elsewhere) from the selected starting point.

### Questionnaire

The questionnaire was broad in scope, covering demographic information, morbidity and mortality, vaccination coverage, sexual and reproductive health, and access to health care. Questions were drawn from instruments used by the investigators for previous surveys of refugee health in collaboration with the Centers for Disease Control and Prevention and the World Health Organization, and translated into Arabic. A standardized questionnaire was used at all sites and the head of household or household representative (husband, wife, widow/mother-in-law or other) was interviewed for information on all members of households. Data was collected both on paper and electronically.

### Analysis

The survey was initially designed to look at a broad range of health issues and this article will concentrate on SRH. The present analysis focuses on two groups of interest among women of reproductive age (15 to 49 years): women who reported being currently pregnant and women who reported having given birth in the previous 12 months. The main reproductive health outcomes of interest were: unplanned pregnancy, antenatal care, skilled birth attendant at time of delivery, and cesarean section. We combined the four sites to explore risk factors and determinants of these four outcomes among the two groups of women, using site and demographic variables as explanatory factors. Note that the question on unplanned pregnancy was not systematically asked for currently pregnant women in Bekaa, and this site was excluded from the analysis of this outcome among currently pregnant women. Sample means and proportions were used to estimate their respective population counterparts. Note also that these survey percentages take into account weighting and refer to the estimated underlying population and not the survey sample. Pearson chi-square tests were used to compare proportions, and generalized linear models were used to calculate odds ratios. Multivariable models for all outcomes were adjusted for demographic variables (age, head of household, education) and information on refugee status (time since departure from home, registration as refugee or IDP, and shelter type). Available obstetric or reproductive health information differed for the two groups of women, and when available the following variables were included in adjusted models: for currently pregnant women: intention to conceive and gestational age; for women having delivered in the previous year: intention to conceive, birth location, and previous cesarean section. All analyses were performed using the survey suite of commands in Stata version 14.1 which takes the sampling methodology into account.

## Results

### Overall results

The total surveyed population consisted of 23,604 individuals in 4444 households including 5925 women between 15 and 49 years of age (“women of childbearing age”, WCA) [Table 1]. A total of 4115 survey respondents reported on 5925 women of childbearing age; the survey respondent was a woman of childbearing age in 58.5% of households (*n* = 2406). There was a median of one WCA per household (IQR 1–2, range 1–7); 72.1% (*n* = 2965) of respondents reported only one WCA and 11.1% (*n* = 455) reported on three or more. Overall, respondents reported that 445 (7.5%) women were pregnant and 760 (12.8%) had given birth within the previous 12 months. For a majority of women in both groups, their pregnancy was said to be not wanted “at the time” or “at all”. This held for 57.5% (*n* = 140; 95% CI 47.9–66.7) of currently pregnant women in Tripoli, Dohuk and Kirkuk and 66.7% (*n* = 496; 95% CI 59.3–73.4) of women who had delivered in the previous year in all four sites.

**Table 1:** Survey population by site

	Bekaa		Tripoli		Duhok		Kirkuk		Total	
	n	%	n	%	n	%	n	%	n	%
Total survey population	6363	27.0	3022	12.8	7628	32.3	6591	27.9	23,604	100%
Women^a^	3282	51.6	1492	49.4	3783	49.6	3228	49.0	11,785	50.0
Women 15–49 years^b^	1548	47.2	830	55.6	1861	49.2	1686	52.2	5925	50.3
Current pregnancy^c^	211	13.7	31	3.7	97	5.2	106	6.29	445	7.5
Delivery in last year^c^	294	19.0	47	5.7	235	12.6	184	10.9	760	12.8

^a^percentage of total population by site; ^b^percentage of women by site ^c^percentage of women 15–49 years by site

Regarding currently pregnant women in all sites, 61.3% (95% CI: 55.3–67.0) (*n* = 270) had had at least one antenatal visit. The presence of a skilled birth attendant at delivery was high (85.4, 95% CI 81.0–88.0) (*n* = 632), as was the proportion of cesarean sections (22.1, 95% CI 18.8–25.4), in women who had delivered in the last year (*n* = 168).

The average age for women of reproductive age was 28 years, and differed significantly by site, ranging from 26.6 in Duhok to 29.7 in Tripoli (*p* < 0.001, Table 2). Overall education was low in this group: 38% (*n* = 325) of household heads reported no formal education, and this ranged from 60.2% in Duhok to 19.5% in Tripoli (*p* < 0.05). Time since departure from home and proportion of people registered as refugees or IDPs with the UNHCR differed by site, reflecting the different stages of the refugee crisis (Fig. 1). Percentages of currently pregnant women and women having given birth in the previous 12 months were highest in Bekaa and lowest in Tripoli [Table 1]. Appropriate antenatal care was lowest in Duhok with just over a third of women accessing antenatal care according to WHO and MSF recommendations, i.e. at least four visits before delivery [Table 2].

**Table 2:** Socio-demographic characteristics and sexual and reproductive health outcomes for women of reproductive age by survey site

Characteristics	Bekaa (*n* = 1548)		Tripoli (*n* = 830)		Duhok (*n* = 1861)		Kirkuk (*n* = 1686)		Total (*n* = 5925)		
	svy %	95% CI	svy %	95% CI	svy %	95% CI	svy %	95% CI	svy %	95% CI	*p*-val^5^
Age (mean)	28.2	27.8–28.5	29.7	28.4–31.1	26.6	26.2–27.0	28.2	27.6–28.9	28.0	27.6–28.4	< 0.001
Head of HH without education	36.9	29.8–44.7	19.5	3.6–61.5	60.2	53.8–66.4	25.6	16.6–37.2	38.2	31.0–46.0	0.038
Time since departure (mean months)	12.5	11.4–13.7	1.2	0–3.4	4.1	4.0–4.3	7.3	6.5–8.2	6.0	5.2–6.7	< 0.001
UNHCR registration	65.5	57.6–72.7	5.9	0.68–36.8	89.6	80.3–94.8	79.4	62.9–89.8	68.3	62.1–74.4	< 0.001
HH size (mean)	5.9	5.7–6.2	5.5	4.3–6.6	6.8	5.7–7.8	5.7	5.4–5.9	6.4	5.6–6.5	0.15
Delivered in survey site region^a^	60.4	50.0–70.0	95.7^#^	84.1–99.0	46.8	40.3–53.4	53.3	44.7–61.7	57.5	37.7–47.4	< 0.001
Previous caesarean section^a^	28.3	22.2–34.4	31.8	15.4–48.2	13.0	16.7–19.4	16.3	11.3–21.3	20.4	16.3–24.4	0.003
Gestational age (mean weeks)^b^	19.9	18.7–21.1	20.0	13.9–26.1	22.9	20.7–25.1	11.9	10.1–13.8	18.9	17.3–20.5	< 0.001
SRH Outcomes											
Unplanned pregnancy^a,c^	67.0	60.0–73.8	61.7	21.3–90.1	68.9	63.3–74.1	70.1	45.4–86.9	66.7	59.3–73.4	0.85
Unplanned pregnancy ^b,c^	–	–	32.2	18.5–50.0	61.9	54.4–68.7	66.0	38.3–85.9	57.5	47.9–66.7	0.07
Antenatal care^b^	62.9	52.0–72.5	87.1	75.4–93.7	44.3	30.6–59.0	64.2	56.1–71.5	61.3	55.3–67.0	< 0.001
Appropriate ANC^b,d^	56.4	46.1–66.1	74.2	63.9–78.6	35.1	24.8–46.9	62.3	54.1–69.8	54.3	49.0–59.6	< 0.001
Skilled birth attendance^a^	93.4	90.8–95.3	91.5	70.3–98.0	71.9	63.1–79.3	89.7	83.1–93.9	85.4	81.0–88.0	< 0.001
Caesarean section^a^	29	23.3–35.5	27.3	9.9–56.1	12.8	9.9–16.4	25.5	20.8–31.0	22.1	18.8–25.4	0.043

^a^among women with delivery in last 12 months (*N* = 294 in Bekaa, *N* = 47 in Tripoli, *N* = 235 in Dohuk, *N* = 184 in Kirkuk, *N* = 760 in total); ^b^ among women currently pregnant (*N* = 211 in Bekaa, *N* = 31 in Tripoli, *N* = 97 in Dohuk, *N* = 106 in Kirkuk, *N* = 445 in total); ^c^Unplanned pregnancy = reported that pregnancy either unwanted or not wanted at this time (aka unmet need for family planning),^d^ at least one visit by 26 weeks- at least 2 visits by 32 weeks and at least 3 visits by 38 weeks- and 4 visits over 38 weeks. ^5^Pearson chi square test for categorical variables- adjusted Wald test for continuous variables (within survey suite of commands). # *n* = 47 women

*Svy* Survey, *CI* Confidence interval, *UNHCR* United Nations High Commissioner for Refugees, *HH* Household, *SRH* Sexual and reproductive health, *ANC* Antenatal care

Unplanned pregnancy did not differ significantly by site amongst women who had delivered in the previous year, and was reported by 66.7% overall. However, amongst currently pregnant women, this proportion was lower in Tripoli than in other sites, where one third of women reported unplanned pregnancy compared to 66% of women who reported the same in Kirkuk (*p* = 0.07).

Although the proportions of deliveries in the presence of a skilled birth attendant was high for women overall, the proportions differed significantly by site, from 93.4% in Bekaa to 71.9% in Duhok, *p* < 0.001 [Table 2].

Nearly a third of women had delivered by cesarean section, with the exception of Dohuk where this figure was 12.8% (95% CI: 9.9–16.4).

### Risk factor analysis

#### Currently pregnant women

In the multivariable model of unplanned pregnancy, only site remained a significant predictor such that women in Kirkuk had higher odds of unplanned pregnancy (OR % 14.08, 95% CI 3.89–50.99) than women in Tripoli (data not available for Bekaa) (Table 3).

**Table 3:** Predictors of unplanned pregnancy and ANC use in currently pregnant women in all sites combined

		Unplanned pregnancy^a^				ANC use			
		OR	95% CI	aOR	95% CI	OR	95% CI	aOR	95% CI
Site	Bekaa	–				1.0		1.0	
	Tripoli	1.0		1.0	1.0	3.99	1.61–9.88	1.53	0.26–9.11
	Dohuk	3.41	1.53–7.59	2.69	0.22–33.61	0.47	0.22–0.99	0.54	0.11–2.59
	Kirkuk	4.08	1.05–15.89	14.08	3.89–50.99	1.06	0.60–1.85	0.96	0.33–2.83
Age (years)	15–19	1.0		1.0	1.0	1.0		1.0	
	20–29	0.94	0.22–4.04	0.97	0.34–2.75	1.21	0.61–2.40	1.14	0.41–3.15
	30–39	1.28	0.38–4.38	1.49	0.54–4.10	0.98	0.41–2.34	1.02	0.32–3.30
	40–49	1.79	0.18–18.17	1.22	0.09–16.28	0.69	0.17–2.91	0.87	0.21–3.54
Head of household education	None	1.0		1.0	1.0	1.0		1.0	
	Primary	0.91	0.57–1.44	1.23	0.66–2.28	1.52	0.90–2.59	0.87	0.49–1.54
	Secondary	0.51	0.18–1.42	0.56	0.19–1.66	3.68	1.48–9.17	4.80	1.70–13.52
	University	0.34	0.08–1.46	0.3	0.09–1.01	4.69	1.47–14.93	2.68	0.98–7.28
Trimester	First (week 1–12)	1.0		1.0	1.0	1.0		1.0	
	Second (week 13–27)	1.21	0.50–2.93	1.2	0.59–2.41	1.11	0.69–1.80	1.05	0.60–1.81
	Third (week 28–36) +	1.37	0.32–5.78	2.09	0.58–7.57	1.16	0.62–2.16	1.51	0.78–2.92
Intention	Planned					1.0		1.0	
	Not now					0.81	0.49–1.33	1.10	0.71–1.71
	Not at all					0.32	0.15–0.69	0.43	0.21–0.89
Shelter type	Own or rent house	1.0		1.0	1.0	1.0		1.0	
	House occupied for free	2.22	0.75–6.57	0.98	0.26–3.69	0.53	0.27–1.04	1.11	0.45–2.73
	Unfinished building	1.72	0.59–5.00	0.79	0.12–5.06	0.26	0.07–0.93	0.74	0.17–3.21
	Other shelter type	2.41	1.17–4.99	1.28	0.28–5.87	0.23	0.12–0.46	0.65	0.16–2.62
	Camp / ITS	4.38	2.32–8.29	0.99	0.12–8.21	0.31	0.16–0.59	0.96	0.39–2.34
Time since leaving home	< 3 months	1.0		1.0	1.0	1.0		1.0	
	3–5 months	4.82	2.44–9.52	1.26	0.14–11.11	0.2	0.07–0.56	0.41	0.11–1.53
	6–11 months	3.71	1.00–13.79	0.45	0.11–1.76	0.31	0.11–0.87	0.29	0.06–1.39
	> 12 months	1.41	0.32–6.32	0.86	0.35–2.13	0.43	0.18–1.03	0.67	0.19–2.43
UNHCR registration		2.53	1.03–6.22	0.93	0.17–4.98	0.64	0.37–1.09	1.45	0.64–3.29

^a^Unplanned pregnancy includes only Tripoli- Dohok- Kirkuk. *ITS* Informal tented settlement, *UNHCR* United Nations High Commissioner for Refugees, *HH* Household, *SRH* Sexual and reproductive health, *ANC* Antenatal care

**Table 4:** Predictors of unplanned pregnancy, skilled birth attendance and caesarean section in women who delivered in the last 12 months in all sites

		Unplanned pregnancy				Skilled birth attendance				Caesarean section			
		OR	95% CI	aOR	95% CI	OR	95% CI	aOR	95% CI	OR	95% CI	aOR	95% CI
Site	Bekaa	1.0		1.0		1.0		1.0		1.0		1.0	
	Tripoli	0.93	0.15–5.72	1.94	0.62–6.11	0.76	0.16–3.59	0.12	0.02–0.67	0.92	0.26–3.24	0.23	0.02–2.73
	Dohuk	1.29	0.85–1.95	0.92	0.33–2.60	0.18	0.11–0.31	0.38	0.09–1.62	0.36	0.24–0.54	0.18	0.08–0.44
	Kirkuk	1.36	0.46–4.03	1.5	0.61–3.68	0.61	0.31–1.20	0.74	0.32–1.73	0.84	0.56–1.25	1.3	0.74–2.28
Age (years)	15–19	1.0		1.0		1.0		1.0		1.0		1.0	
	20–29	1.39	0.75–2.58	1.16	0.56–2.41	0.61	0.24–1.58	0.75	0.26–2.16	1.25	0.78–2.01	1.15	0.59–2.25
	30–39	1.91	0.84–4.36	1.63	0.80–3.31	0.35	0.15–0.83	0.43	0.15–1.27	1.14	0.63–2.04	1.19	0.55–2.56
	40–49	2.79	0.98–7.93	1.97	0.72–5.36	1.06	0.31–3.61	1.23	0.23–6.45	1.45	0.54–3.85	1.16	0.38–3.50
Head of HH educ.	None	1.0		1.0		1.0		1.0		1.0		1.0	
	Primary	0.74	0.48–1.14	0.79	0.53–1.17	3.13	1.70–5.77	1.65	0.79–3.41	2.23	1.38–3.59	1.04	0.54–2.00
	Secondary	0.52	0.25–1.09	0.60	0.29–1.24	3.81	1.28–11.36	2.1	0.58–7.58	2.03	1.04–3.96	1.03	0.40–2.70
	University	0.42	0.16–1.10	0.44	0.19–0.98	6.19	1.28–29.90	3.17	0.68–14.80	2.1	0.99–4.49	1.05	0.32–3.45
Intention	Planned					1.0		1.0		1.0		1.0	
	Not now					0.82	0.37–1.84	1.07	0.46–2.46	0.61	0.41–0.91	0.47	0.26–0.88
	Not at all					0.61	0.27–1.40	1.2	0.46–3.14	0.93	0.53–1.61	0.91	0.51–1.60
Birth in survey site region		0.66	0.40–1.09	0.71	0.47–1.06	4.07	2.40–6.88	3.55	1.94–6.49	1.84	1.34–2.53	1.91	1.29–2.84
Previous CS		1.26	0.68–2.34	1.45	0.84–2.52	2.07	1.14–3.75	1.35	0.66–2.74	16.23	9.29–28.36	17.54	8.89–34.59
Shelter type	Own/rent house	1.0		1.0		1.0		1.0		1.0		1.0	
	House for free	0.95	0.35–2.55	0.89	0.34–2.32	0.24	0.07–0.76	0.59	0.11–3.03	0.74	0.35–1.57	1.09	0.46–2.61
	Unfinished bldg.	2.25	0.67–7.51	2.14	0.63–7.25	0.21	0.07–0.61	0.42	0.12–1.47	0.44	0.24–0.81	1.4	0.67–2.94
	Camp / ITS	1.42	0.62–3.23	1.59	0.68–3.69	0.2	0.07,0.62	0.56	0.15–2.13	0.42	0.21–0.84	1.48	0.73–3.01
	Other	1.33	0.54–3.27	1.13	0.52–2.43	0.97	0.14–6.50	0.67	0.19–2.35	0.68	0.39–1.18	0.71	0.46–1.11
Time since leaving home	< 3 months	1.0		1.0		1.0		1.0		1.0		1.0	
	3–5 months	1.64	0.35,7.77	1.79	0.53–6.12	0.11	0.03–0.44	0.12	0.01–1.62	0.4	0.19–0.85	0.71	0.32–1.59
	6–11 months	1.51	0.29,7.84	1.85	0.66–5.22	0.35	0.09–1.42	0.16	0.02–1.30	0.75	0.36–1.57	0.38	0.21–0.70
	> 12 months	1.41	0.30,6.60	2.32	0.68–7.94	0.53	0.18–1.52	0.16	0.03–0.84	0.65	0.36–1.18	0.32	0.18–0.57
UNHCR registration		1.19	0.61–2.30	0.86	0.48–1.51	0.95	0.52–1.72	1.94	0.99–3.79	0.62	0.36–1.06	0.87	0.54–1.40

*ITS* Informal tented settlement, *UNHCR* United Nations High Commissioner for Refugees, *HH* Household, *SRH* Sexual and reproductive health

In terms of antenatal care, head of household education and intention to conceive were predictive of at least one ANC visit in multivariable analysis. Women living in a household where the head of household had secondary education had greater odds of ANC compared to those with no education (OR: 4.80, 95% CI 1.7–13.5). And women who reported that their pregnancy was not intended (“not at all”) had less than half the odds of reporting any ANC than women who said their pregnancy was planned (OR 0.43 95% CI 0.21–0.89).

### Women who had delivered in the previous 12 months

For women who had delivered in the previous year, living with a head of household who had university education was associated with lower odds of unplanned pregnancy (OR 0.44, 95% CI 0.19–0.98) than a head of household with no education (Table 4).

Location of delivery was a significant predictor of skilled birth attendance in multivariable analysis, such that women who had given birth in the survey site (i.e. the area to which they had been displaced) had more than triple the odds of presence of a skilled attendant compared to those who had given birth in their home region (OR 3.55, 95% CI 1.94–6.49). Site was associated with the presence of a skilled birth attendant, such that women in Tripoli had much lower odds of presence of a skilled birth attendant at delivery (OR 0.12 95% CI 0.02–0.67) than women in Bekaa (no evidence for a difference in the other sites).

The proportion of women delivering by cesarean section was high overall at 22.1% across the four sites, and factors associated with a higher odds of a cesarean a previous cesarean (OR 17.54 95% CI 8.89–34.59) and delivery at survey site compared to home region (1.91, 95% CI 1.29–2.84).

## Discussion

The results of this survey highlight three components of SRH requiring improvement: 1) proportions of unplanned pregnancies are overall high; 2) access to antenatal care is suboptimal; 3) proportions of cesarean sections are significantly above the recommended limits.

We shall discuss each of these points separately.

### High proportions of unplanned pregnancies

The question of unplanned pregnancy remains a sensitive issue. Worldwide, around 40% of pregnancies are thought to be unintended [16]. In 2009 it was estimated that one in three pregnancies was unintended in the Middle East and North Africa region [3]. However, many women may be reluctant to state that their pregnancy was unplanned and/or may change their view once the child is born. Thus, data on unintended pregnancies cannot be considered as thoroughly reliable. It is clear that Syrian, Iraqi and vulnerable Lebanese women have, at best, experienced ruptures in contraceptive stocks or, at worst, have no access to modern contraceptive methods for prolonged periods. It is known that unintended pregnancies are associated with a range of physical and psychological risks including reduced access to antenatal care, greater risk-taking behaviors (such as smoking), low birth weight for the baby, and even maternal death [17]. Indeed in our survey, we found that women who did not intend to get pregnant were less likely to access ANC at all. Risk factors for unplanned pregnancy in our survey were linked to site, with higher proportions being reported in Iraq. Why this is so is not clear and would require further investigation. Sexual violence, for instance, is more prevalent in conflict/displacement situations than in stable circumstances and this can contribute to higher unintended pregnancy rates. There have been numerous reports of sexual violence in the present Syrian crisis, both in Syria and in host countries, even given the strong cultural reluctance and fear to talk about such subjects [18–20]. Another fact to consider is that early marriage has emerged as a “coping strategy” for families with young girls [21]: it is hoped that married teenage women will be thus better protected and provided for. However, early marriage can be associated with poorer knowledge of family planning methods, higher maternal risks, and poorer pregnancy outcomes [22, 23]. Finally, beyond pregnancies occurring from unwanted sexual relations, it is not clear whether more women dread becoming pregnant in a refugee context or whether some women are having children in order to “compensate” for lives lost during the war; this ambiguity of attitude towards pregnancy has been noted in many other contexts [24]. Unfortunately, we were not able to examine these important factors in the context of our survey.

### Suboptimal access to antenatal care

After having recommended four antenatal visits for pregnant women, the WHO speaks now of at least eight “contacts” with a health care provider during pregnancy with a view to preventing health problems for both mother and child [25]. Prenatal care includes tetanus vaccination, screening for and treatment of infections, and identification of early warning signs of complications. Reaching this goal in a refugee situation can be challenging. However, lack of antenatal care is associated with a higher risk of complex deliveries and health issues for both mother and child [26, 27]. In our survey, we saw that coverage by at least one antenatal care visit was highest in Tripoli (nearly 75%) – which was the first location where MSF intervened – and lowest in Dohuk (35%). Women who had planned their pregnancy were more likely to access ANC, than those who stated their pregnancy was unintended, underscoring the need for family planning. Clearly, the target of eight visits with a health care provider during pregnancy is far from being met, but even if one considers the former WHO target of four antenatal care visits (which is more realistic in this context), coverage remains suboptimal. This is interesting given that a similar study conducted soon after ours found that 89% of Syrian women in Lebanon had sought ANC with an average of six visits [25].

### High proportions of cesarean sections

Cesarean section rates in Syria and Lebanon were high before the Syrian crisis, with estimates of up to 35% in Lebanon and 45% in Syria [28]. Cesarean section is not a minor event and carries risks for both mother and child. The WHO estimates that between 5 and 15% of deliveries at a population level necessitate the procedure, and states that there is no evidence of benefit to the mother or child of a medically unnecessary procedure [29, 30]. In the present context, with an average of 22.1% of cesarean sections for women having delivered in the previous year across all four sites, we are clearly over the limit. Another study in Lebanon found that around one-third of Syrian women had had a cesarean section [31]. The question is: are they medically necessary? In our survey, the strongest predictor of a cesarean section was a previous cesarean section. It has been reported that currently in Syria some health care professionals suggest cesarean section to expectant mothers so that, in unstable conditions, they can plan their delivery. Several studies have found higher cesarean section rates in conflict settings [28, 32]. A survey in 2013 found that up to 41% of deliveries in the Bekaa Valley were by cesarean section, in spite of the fact that most women said they preferred vaginal birth [28]. We found a lower percentage of cesarean sections (29%) in the same area at around the same time. Medical considerations aside, the UNHCR covers 75% of costs associated with delivery, such that when there is a cesarean section families may have to pay up to the equivalent of 500 euros for the extra 25% [33].

### Limitations & generalizability

Our study has several limitations. To begin with, the uncertainty surrounding the use of probability sampling methods in the second stage of sampling in Bekaa, Dohuk and Kirkuk, as well as for the selection of the starting household in Tripoli, suggests that the sampled respondents may not be representative of the refugees or IDPs living in these sites, and results may be biased. Secondly, our survey was not designed specifically to address reproductive health issues, but was a general health assessment at one point in time in a constantly evolving and complex environment. Third, we identified many differences among survey sites, but were unable fully to explore the reasons for these differences. At a programmatic level, we could not examine how differences in service provision would have affected service use between sites. Also we lacked information on important individual level determinants of our outcomes, e.g. we did not include questions on parity, miscarriage or stillbirth (for which there is a known lack of data [1]), marital status, or age at marriage. It would have likewise been valuable to address questions of sexual violence (including intra-partner violence) both as an outcome in itself and as a determinant of service use; however, this subject is associated with such stigma (and even danger for the woman admitting to it) [18], that to include it in the questionnaire would probably have been counterproductive. Also, we did not delve into the issue of depression/anxiety during pregnancy and postpartum which, in an insecure setting, might affect more women than in non-conflict circumstances [34]. A final but important limitation to our study is the fact that the respondent to the health assessment questionnaire was not always the woman herself (justified by the fact that the survey was not restricted to SRH). Only in the case of households where the survey respondent was a woman of childbearing age and where there was only one woman of childbearing age can we be sure that the woman was reporting on herself, which occurred for 1782 (30%) of women. While this would probably not have affected the reporting of outcomes such as birth and skilled delivery to an important degree, it may well have affected questions relating to pregnancy and especially to unintended pregnancy, and might have biased also the results of risk factor analysis. The exclusion of the Bekaa site from the analysis of unintended pregnancy among currently pregnant women may also limit the generalizability of the results on unmet need for family planning among currently pregnant Syrian refugees. Despite these limitations, this is to our knowledge one of the only studies to concentrate on reproductive health needs of Syrian and Iraqi displaced women using systematic survey methods and a large sample. Thus we feel our main findings are robust and likely apply to Syrian and Iraqi displaced women in other host countries as well as to vulnerable Lebanese.

## Conclusions

Since the 1990s, sexual and reproductive health has been increasingly integrated into humanitarian responses [34, 35]. The minimal initial service package for reproductive health introduced a set of priorities for agencies providing medical care in emergencies in the late 1990s and included making sure that contraceptives and condoms are freely available, and providing clean delivery kits and a 24/7 referral system for obstetric emergencies [35]. While aid agencies provide care according to international standards, they often operate within host country national guidelines, which can particularly affect sensitive issues such as family planning and delivery care [36].

Problems associated with reproductive health are being compounded by a series of interacting risk factors including poor access to family planning, unintended pregnancies, suboptimal access to antenatal care, and over-recourse to cesarean sections. It would be interesting to conduct further research examining trends over time and looking notably at Syrian women in other host countries. But even in the absence of this information, as the refugee crisis evolves, it is clear that women have real needs which – if unmet – can critically affect their physical health and psychosocial wellbeing as well as that of their children. It is not unlikely that the challenges we describe here can be observed also in similar migrant populations [37, 38]. While more data may be desirable in order to fine-tune intervention strategies, it is crucial to place sexual and reproductive health needs – and in particular adequate antenatal care, family planning, and safe termination of pregnancy – high on the agenda of aid agencies. This is all the more true today, in a context where massive numbers of Syrian refugees maybe returning (voluntarily or involuntarily) to their home country with unknown levels of access to care.

## Data Availability

The data can be made available upon reasonable demand.

## Abbreviations

ANC: Antenatal Care
IDP: Internally Displaced Person
MSF: Médecins Sans Frontières
NGO: Non Governmental Organization
SRH: Sexual Reproductive Health
ToP: Termination of Pregnancy
UN: United Nations
UNHCR: United Nations High Commission for Refugees
